# Mast Cells Density Positive to Tryptase Correlate with Microvascular Density in both Primary Gastric Cancer Tissue and Loco-Regional Lymph Node Metastases from Patients That Have Undergone Radical Surgery

**DOI:** 10.3390/ijms17111905

**Published:** 2016-11-15

**Authors:** Michele Ammendola, Rosario Sacco, Valeria Zuccalà, Maria Luposella, Rosa Patruno, Pietro Gadaleta, Nicola Zizzo, Cosmo Damiano Gadaleta, Giovambattista De Sarro, Giuseppe Sammarco, Mihai Oltean, Girolamo Ranieri

**Affiliations:** 1 Department of Medical and Surgical Sciences, Clinical Surgery Unit, University “Magna Graecia” Medical School, Viale Europa, Germaneto, 88100 Catanzaro, Italy; sacco@unicz.it (R.S.); sammarco@unicz.it (G.S.); 2 Surgery Unit, National Cancer Research Centre, Istituto Tumori “Giovanni Paolo II”, Viale Orazio Flacco 65, 70124 Bari, Italy; 3 Pathology Unit, “Pugliese-Ciaccio” Hospital, Viale Pio X, 88100 Catanzaro, Italy; valezy@libero.it; 4 Cardiovascular Disease Unit, “San Giovanni di Dio” Hospital, 88900 Crotone, Italy; marilyn_luposella@live.it; 5 Chair of Pathology, Veterinary Medical School, University “Aldo Moro” of Bari, Via Casamassima, 70010 Bari, Italy; rosavet@libero.it (R.P.); nicola.zizzo@uniba.it (N.Z.); 6 Diagnostic and Interventional Radiology Unit with Integrated Section of Translational Medical Oncology, National Cancer Research Centre, Istituto Tumori “Giovanni Paolo II”, viale Orazio Flacco 65, 70124 Bari, Italy; gadaleta5@libero.it (P.G.); c.gadaleta@oncologico.bari.it (C.D.G.); iroran@tiscalinet.it (G.R.); 7 Department of Health Science, Clinical Pharmacology and Pharmacovigilance Unit and Pharmacovigilance’s Centre Calabria Region, University of Catanzaro “Magna Graecia” Medical School, Viale Europa, Germaneto, 88100 Catanzaro, Italy; desarro@unicz.it; 8 The Institute for Clinical Sciences, Department of Transplantation, University Hospital, Sahlgrenska Academy at the University of Gothenburg, 41345 Gothenburg, Sweden; mihai.oltean@surgery.gu.se

**Keywords:** angiogenesis, mast cells, tryptase, prognostic factor, gastric cancer, therapy

## Abstract

Mast Cells (MCs) play a role in immune responses and more recently MCs have been involved in tumoral angiogenesis. In particular MCs can release tryptase, a potent in vivo and in vitro pro-angiogenic factor via proteinase-activated receptor-2 (PAR-2) activation and mitogen-activated protein kinase (MAPK) phosphorylation. MCs can release tryptase following c-Kit receptor activation. Nevertheless, no data are available concerning the relationship among MCs Density Positive to Tryptase (MCDPT) and Microvascular Density (MVD) in both primary gastric cancer tissue and loco-regional lymph node metastases. A series of 75 GC patients with stage T_2–3_N_2–3_M_0_ (by AJCC for Gastric Cancer Seventh Edition) undergone to radical surgery were selected for the study. MCDPT and MVD were evaluated by immunohistochemistry and by image analysis system and results were correlated each to other in primary tumor tissue and in metastatic lymph nodes harvested. Furthermore, tissue parameters were correlated with important clinico-pathological features. A significant correlation between MCDPT and MVD was found in primary gastric cancer tissue and lymph node metastases. Pearson *t*-test analysis (*r* ranged from 0.74 to 0.79; *p*-value ranged from 0.001 to 0.003). These preliminary data suggest that MCDPT play a role in angiogenesis in both primary tumor and in lymph node metastases from GC. We suggest that MCs and tryptase could be further evaluated as novel targets for anti-angiogenic therapies.

## 1. Introduction

Mast Cells (MCs) can play a role in tumor angiogenesis and their involvement has been demonstrated in several animal and human malignancies [1,2]. MCs can secrete classical pro-angiogenic factors, including Vascular Endothelial Growth Factor (VEGF), Fibroblast Growth Factor-2 (FGF-2), Thymidine Phosphorylase (TP) as well as tryptase, a non-classical pro-angiogenetic factor [3,4,5,6,7]. From them, Tryptase is the most abundant factor contained in MCs secretory granules and it can be released by several mechanisms including c-Kit receptor activation. Interestingly, it has been demonstrated that tryptase induces in vitro endothelial cell (ECs) proliferation in a matrigel assay and displayed in vivo capillary growth in the chick embryo chorioallantoic membrane, which was suppressed by tryptase inhibitors [8]. From a biological point of view, the signalling induced by tryptase can be mediated via protease-activated receptor-2 (PAR-2) expressed on ECs, that in turn lead to ECs proliferation forming angiogenesis [9,10,11,12,13,14,15,16,17,18,19,20,21,22,23,24,25,26,27].

So far, few data have been published on the relationship among MCs density positive to tryptase (MCDPT), and microvascular density (MVD) in both primary gastric cancer (GC) tissue and loco-regional lymph node metastases (LRLNM) [6,28,29,30,31,32].

In GC, the presence of lymph node metastases is one of the most important determinants of prognosis and tumor node metastases (TNM) is the most commonly used staging system [33].

In this pilot study, we analyzed by immunohistochemistry and image analysis system MCDPT and MVD in both primary GC tumor tissue (PGCTT) and LRLNM from 75 patients undergoing radical surgery. The correlation between the studied parameters and the main clinico-pathological features has been also performed.

## 2. Results

**Figure 1:**
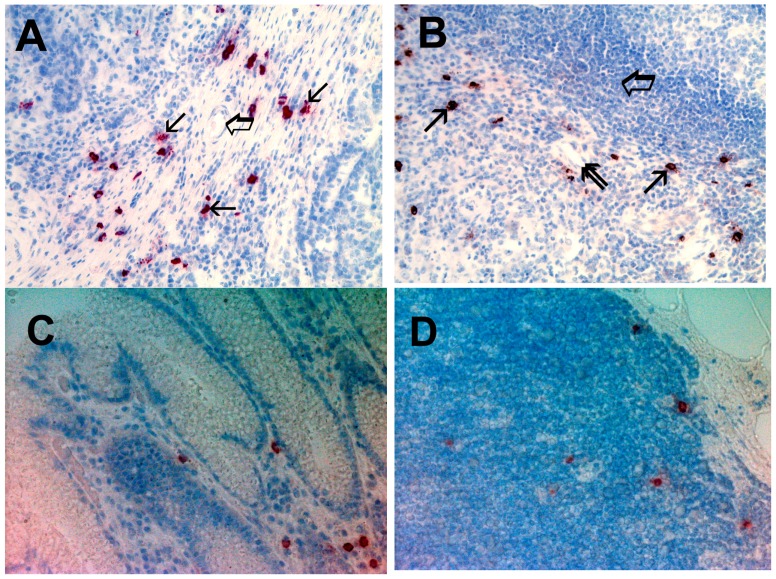
(**A**) Primary gastric cancer tissue section immunostained with the anti-tryptase antibody. Single arrows indicate single red stained mast cells. Big arrow indicates a blood microvessel with a red blood cell in its lumen. Magnification 40× (0.19 mm^2^ area); (**B**) Lymph node metastases from primary gastric cancer tissue section immunostained with the anti-tryptase antibody. Single arrows indicate single red stained mast cells. The big arrow indicates residual lymphocytes, double arrow indicates a single microvessel. Magnification 40× (0.19 mm^2^ area); (**C**) Adjacent normal gastric tissue section immunostained with the anti-tryptase antibody; and (**D**) Normal lymph node section immunostained with the anti-tryptase antibody.

**Figure 2:**
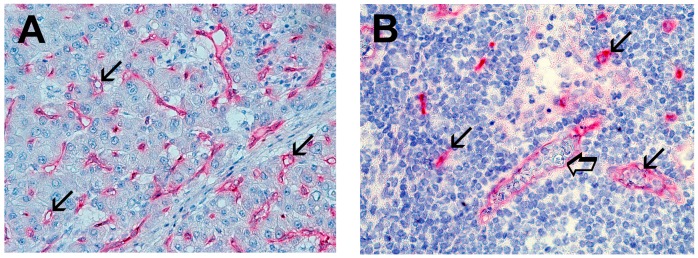

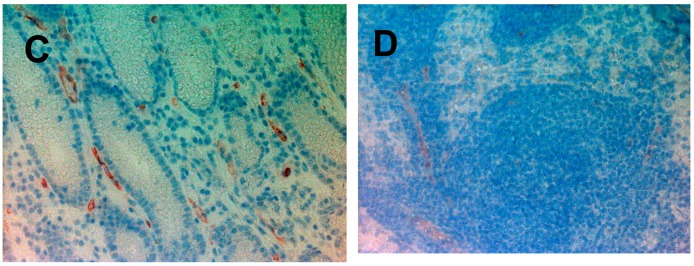
(**A**) Primary gastric cancer tissue section immunostained with the anti-CD34 antibody. Single arrows indicate single red stained microvessels. Magnification 40× (0.19 mm^2^ area); (**B**) Lymph node metastases from primary gastric cancer tissue section immunostained with the anti-CD34 antibody. Single arrows indicate single red stained microvessels. The big arrow indicates blood vessels containing many metastatic gastric cancer cells. Magnification 40× (0.19 mm^2^ area); (**C**) Adjacent normal gastric tissue section immunostained with the anti-CD34 antibody; and (**D**) Normal lymph node section immunostained with the anti-CD34 antibody.

Immunohistochemical staining by using the antibodies anti-tryptase showed red-brown positive to tryptase MCs. MCs showed round or ovoidal shape and heterogeneous size also. In Figure 1, single small arrows indicate single red-brown immunostained MCs in: A, primary gastric cancer tissue; B, metastatic lymph node; C, adjacent normal gastric tissue and D, normal lymph node. In particular, in Figure 1A, several immunostained MCs near blood vessel, with a perivascular position, are visible with the blood vessel indicated by a big arrow. MCDPT (Figure 1A–D, respectively) was assessed counting each single immunostained mast cell clearly separated each to other at 40× magnification. With special reference to the microvessels, they were identified by the primary anti-CD34 antibody and as red immunostained structure often presenting a small lumen but sometime without a clearly visible lumen. Interestingly, the blue stained nucleus of endothelium was often distinguished in the context of the red immunostained endothelium. In detail, in Figure 2, small arrows indicates microvessels: A, in primery gastric cancer tissue; B, in metastatic lymph node; C, in adjacent normal gastric tissue and D, in normal lymph node. MVD was detected counting each single immunostained endothelial cell or each immunostained endothelial cell clearly separated from adjacent microvessels, tumor cells, and other connective tissue elements.

In LRLNM, the mean value of MCDPT was: 10.18 ± 4.66 in T_2_N_2_M_0_, 12.52 ± 3.32 T_2_N_3_M_0_, 11.89 ± 4.46 in T_3_N_2_M_0_, 13.20 ± 4.95 in T_3_N_3_M_0_. The mean value of MVD was: 23.37 ± 9.07 in T_2_N_2_M_0_, 24.08 ± 8.11 T_2_N_3_M_0_, 24.75 ± 8.59 in T_3_N_2_M_0_, 25.03 ± 8.98 in T_3_N_3_M_0_.

According to the AJCC classification, the mean value ± standard deviation regarding MCDPT and MVD in PGCTT and LRLNM are reported in Table 1. In PGCTT the mean value of MCDPT was: 12.47 ± 4.32 in T_2_N_2_M_0_, 11.67 ± 3.79 in T_2_N_3_M_0_, 13.23 ± 3.92 in T_3_N_2_M_0_, 14.44 ± 4.72 in T_3_N_3_M_0_. The mean value of MVD was: 27.34 ± 8.97 in T_2_N_2_M_0_, 28.22 ± 9.12 in T_2_N_3_M_0_, 28.23 ± 8.88 in T_3_N_2_M_0_, 28.45 ± 9.31 in T_3_N_3_M_0_.

As control cases, far from inflammation, the mean value of MCDPT and MVD in adjacent normal gastric tissue was 4.12 ± 3.10 and 10.40 ± 7.95, respectively. In normal lymph nodes, the mean value of MCDPT and MVD was 5.14 ± 2.63 and 9.39 ± 2.17, respectively.

Based on the obtained data, we showed that tryptase positive mast cells increase between normal tissue and tumor tissue and this correlation also holds for blood vessels.

**Figure 3:**
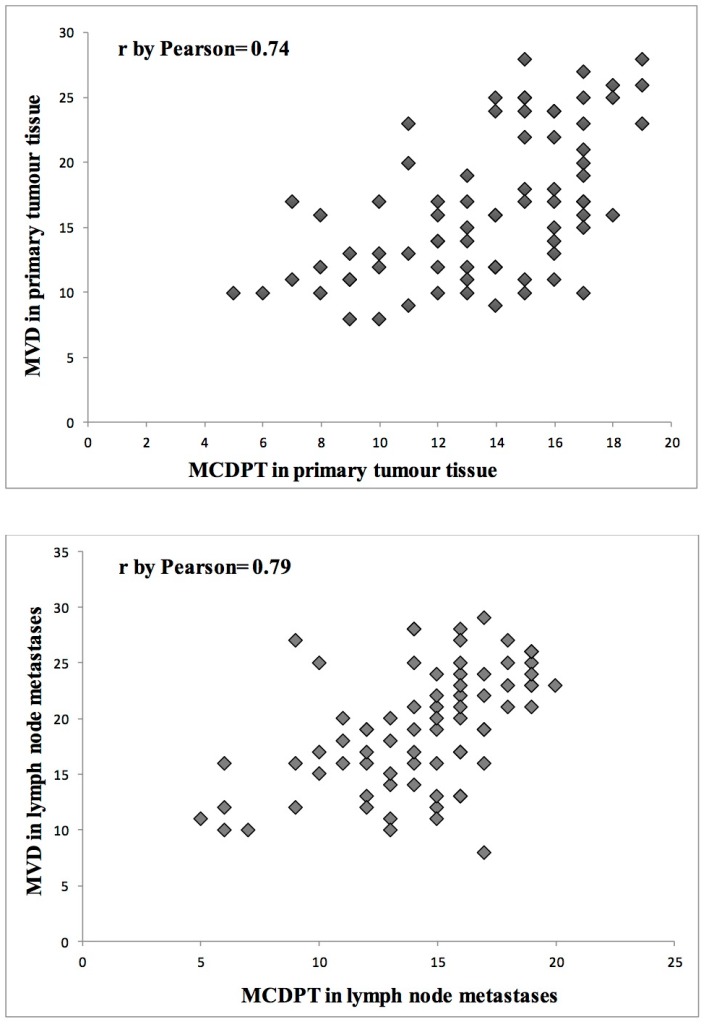
Correlation between MCDPT and MVD in PGCTT (*r* = 0.74, *p* = 0.003) and LRLNM (*r* = 0.79, *p* = 0.001).

With special regard to tumor tissue and lymph node metastases, a significant correlation was found between MCDPT and MVD in PGCTT (*r* = 0.74, *p* = 0.003) and LRLNM (*r* = 0.79, *p* = 0.001) (Figure 3). No correlation concerning MCDPT, MVD, and patient sex was found.

## 3. Discussion

A large body of evidence supports the central role of angiogenesis in GC development and progression but few data regarding the role of MCs in GC angiogenesis have been published [34,35,36]. In particular, in a study performed by Mukherjee et al. [37], the authors studied MCs density in tissue from patients with gastric ulcers and in tissue from GC patients. In the above study the histochemical method of toluidine blue was employed to identify and count MCs density. Data from this study indicated that MCs density in benign gastric ulcers and in cancers was much higher than the control and correlated with angiogenesis.

Ribatti et al. [30] studied tumor samples from GC patients by mean of immunohistochemistry employing anti-tryptase and anti-chymase antibodies to stain MCs. In this study, a correlation between MVD and tryptase and chymase-positive MCs was found suggesting the involvement of MCs in neovascularization. 

Recent published pilot data from our group indicated that MCDPT may induce angiogenesis in bone metastases from gastric cancer patients suggesting that MCDPT is able to stimulate angiogenesis in both primary tumor tissue and metastatic tumor site [21].

To this regard, it is important to underline that MCs can stimulate neovascularization releasing mainly tryptase stored in their secretory granules [38,39].

In the tumor microenvironment, MCs can be activated in different ways including c-kit receptor stimulation and phosphorylation by its ligand the stem cell factor, IgE-dependent mechanism mediated by T lymphocytes-MCs interaction and other microenvironmental stimuli [40,41]. After activation, intensive or piecemeal degranulation of secretory granules occurs depending on MCs activation mechanism, and MCs derived tryptase is released in tumor microenvironment stimulating angiogenesis [7,42,43,44,45,46]. In a pre-clinical model tryptase induces in vitro ECs proliferation in a matrigel assay and displayed in vivo capillary growth in the chick embryo chorioallantoic membrane, which was suppressed by tryptase inhibitors. Tryptase is an agonist of PAR-2 in vascular ECs that stimulates their proliferation. Signaling via PAR-2 on ECs elicits activation of the major members of the MAPK phosphorylation family and contributes to proliferation of ECs and angiogenesis [23,24,25,26,27,28,29,30,31,32,47,48,49,50,51].

Our data demonstrated an association between MCDPT and MVD supporting the central role of tryptase as a main pro-angiogenic factor in PGCTT and interestingly also in LRLNM [52,53,54,55,56].

Our results agree with very recent published research from Micu et al. [57]. These authors demonstrated the positive correlations between the density of tryptase positive mast cells and that of new blood vessels in primary gastric cancer tissue. Interestingly, in the above research, new blood vessels were identified with the anti-CD105 antibody, the marker of endoglin, that is preferentially and selectively expressed on the new blood vessels.

Further studies could be performed employing the anti-CD105 antibody, as a more specific marker of tumor angiogenesis in both primary gastric cancer tissue and lymph node metastasis also to confirm our data obtained with the anti-CD34 pan-endothelial marker. From an anatomical point of view, obtained results indicated that MCDPT are located in a perivascular position. For this reason, we think that some of MCs degranulation products, like tryptases, stay in the stroma of the tumor microenvironment, others go through blood and lymphatic vessels with metastatic dissemination effects.

Based on this background, will be possible to design clinical trials with novel anti-angiogenetic therapies targeting MCs degranulation by mean of c-Kit-R tyrosine kinase inhibitors (e.g., masitinib) or inhibiting tryptase by mean of gabexate mesilate or nafamostat mesilate [58,59,60,61,62,63]. Finally, MCDPT could be evaluated as a possible predictive biomarker able to select patients candidable to novels anti-angiogenic strategies.

## 4. Materials and Methods

### 4.1. Study Population

A series of 75 GC patients diagnosed with preoperative gastric endoscopy were selected to undergo curative resection. The surgical approaches used were open total and sub-total gastrectomy with D2 lymph node dissection. Patients were staged as T_2–3_N_2–3_M_0_ (by AJCC for Gastric Cancer Seventh Edition) according to the American Joint Committee on Cancer Seventh Edition (AJCC-TNM) classification [33,64,65]. None had distant metastases on computed tomography (CT) of the thorax, abdomen, and pelvis. All patients in this study had adenocarcinomas. The clinico-pathological features of the patients are summarized in Table 2. Full ethical approval and signed consent from individual patients was obtained. The study was conducted in accordance with the Declaration of Helsinki, and the protocol was approved by the Ethics Committee of the “Mater Domini” Hospital, “Magna Graecia” University, Catanzaro (2011.61; 13 December 2011).

### 4.2. Immunohistochemistry

For the evaluation of MCDPT and MVD a three-layer biotin-avidin-peroxidase system was utilized [66]. Briefly, 6 μm-thick serial sections of formalin-fixed and paraffin-embedded tumor samples, adjacent normal gastric tissue, lymph node metastases, and normal lymph node were cut. Sections were first deparaffinized and then, for antigen retrieval, sections were microwaved at 500 W for 10 min, after which endogenous peroxidase activity was blocked with 3% hydrogen peroxide solution [21]. Tumor sections were incubated with the following primary antibodies: anti-tryptase (clone AA1; Dako, Glostrup, Denmark) diluted 1:100 for 1 h at room temperature (for MCs identification) and anti-CD34 antibody (QB-END 10; Bio-Optica, Milan, Italy) diluted 1:50 for 1 h at room temperature as a pan-endothelial marker. The bound antibody was visualized using a biotinylated secondary antibody, an avidin-biotin peroxidase complex and liquid permanent red (LPS, K0640, Dako). Nuclear counterstaining was performed with Gill’s haematoxylin No.2 (Polysciences, Warrington, PA, USA). The primary antibody was omitted in negative controls.

### 4.3. Morphometrical Assay

Light microscopy integrated with an image analysis system (AXIO, Scope A1, ZEISS, Gottingen, Germany) was utilized [65]. In sections of primary tumor tissue, lymph node metastases, adjacent gastric normal tissue, and normal lymph nodes, immunostained areas (hot spots) were selected at 10× magnification. Next, MCDPT (Figure 1A–D, respectively) was assessed counting each single immunostained mast cell clearly separated each to other at 40 × magnification. With special regard to MVD, we considered a microvessel each single immunostained endothelial cell or each immunostained endothelial cell with or without a lumen, clearly separated from adjacent microvessels, tumor cells, and other connective tissue elements (Figure 2A–D, respectively) at 40× magnification. To define the evaluated microscopic fields at 40× magnification (ocular lens 10× and objective lens 40×), the corresponding 0.19 mm^2^ area for each field was measured by semi-automated modality using the program of the above image analysis system.

### 4.4. Statistical Analysis

Mean values ± Standard Deviation (SD) of all the evaluated tissue parameters are reported in Table 1. Correlations between MCDPT and MVD were calculated using Pearson’s (r) analysis. Correlations among all the analyzed parameters and the main clinico-pathological features listed in Table 2 were performed by the Chi-square test (χ^2^). *p* < 0.05 was considered significant. All statistical analyses were performed with the SPSS statistical software package (SPSS, Inc., Chicago, IL, USA).

## Figures and Tables

**Table 1:** MCDPT and MVD means ± standard deviations as a function of GC tumor tissue, respectively.

Tissue	MCDPT 40× (0.19 mm^2^)	MVD 40× (0.19 mm^2^)
Primary Tumor		
T_2_N_2_M_0_	12.47 ± 4.32	27.34 ± 8.97
T_2_N_3_M_0_	11.67 ± 3.79	28.22 ± 9.12
T_3_N_2_M_0_	13.23 ± 3.92	28.23 ± 8.88
T_3_N_3_M_0_	14.44 ± 4.72	28.45 ± 9.31
Lymph Node Metastases		
T_2_N_2_M_0_	10.18 ± 4.66	23.37 ± 9.07
T_2_N_3_M_0_	12.52 ± 3.32	24.08 ± 8.11
T_3_N_2_M_0_	11.89 ± 4.46	24.75 ± 8.59
T_3_N_3_M_0_	13.20 ± 4.95	25.03 ± 8.98
Normal Tissue	4.12 ± 3.10	10.40 ± 7.95
Normal Lymph Nodes	5.14 ± 2.63	9.39 ± 2.17

**Table 2:** Clinico-pathological features of patients (*n* = 75).

Age	N
≤65	30
≥65	454
Gender	
Male	42
Female	33
Tumor Site	
Cardia	11
Lesser curvature	7
Greater curvature	9
Body and fundus	23
Pyloric area	25
TNM by AJCC Stage and Type by Lauren Classification	
T_2–3_N_2_M_0_	46
T_2–3_N_3_M_0_	29
Intestinal type	44
Diffuse type	31
Histologic Grade	
G1–G2	55
G3	20

## References

[1] Gulubova M., Vlaykova T. (2009). Prognostic significance of mast cell number and microvascular density for the survival of patients with primary colorectal cancer. J. Gastroenterol. Hepatol..

[2] Marech I., Ammendola M., Sacco R., Capriuolo G.S., Patruno R., Rubini R., Luposella M., Zuccalà V., Savino E., Gadaleta C.D. (2014). Serum tryptase, mast cells positive to tryptase and microvascular density evaluation in early breast cancer patients: Possible translational significance. BMC Cancer.

[3] Marech I., Ammendola M., Gadaleta C., Zizzo N., Oakley C., Gadaleta C.D., Ranieri G. (2014). Possible biological and translational significance of mast cells density in colorectal cancer. World J. Gastroenterol..

[4] Ammendola M., Sacco R., Sammarco G., Donato G., Montemurro S., Ruggieri E., Patruno R., Marech I., Cariello M., Vacca A. (2014). Correlation between serum tryptase, mast cells positive to tryptase and microvascular density in colo-rectal cancer patients: Possible biological-clinical significance. PLoS ONE.

[5] Ammendola M., Sacco R., Sammarco G., Donato G., Zuccalà V., Romano R., Luposella M., Patruno R., Vallicelli C., Verdecchia G.M. (2013). Mast Cells Positive to Tryptase and c-Kit Receptor Expressing Cells Correlates with Angiogenesis in Gastric Cancer Patients Surgically Treated. Gastroenterol. Res. Pract..

[6] Ammendola M., Sacco R., Donato G., Zuccalà V., Russo E., Luposella M., Vescio G., Rizzuto A., Patruno R., de Sarro G. (2013). Mast cell positivity to tryptase correlates with metastatic lymph nodes in gastrointestinal cancer patients treated surgically. Oncology.

[7] Marech I., Leporini C., Ammendola M., Porcelli M., Gadaleta C.D., Russo E., de Sarro G., Ranieri G. (2015). Classical and non-classical proangiogenic factors as a target of antiangiogenic therapy in tumor microenvironment. Cancer Lett..

[8] Ribatti D., Ranieri G., Nico B., Benagiano V., Crivellato E. (2011). Tryptase and chymase are angiogenic in vivo in the chorioallantoic membrane assay. Int. J. Dev. Biol..

[9] Blair R.J., Meng H., Marchese M.J., Ren S., Schwartz L.B., Tonnesen M.G., Gruber B.L. (1997). Human mast cells stimulate vascular tube formation. Tryptase is a novel, potent angiogenic factor. J. Clin. Investig..

[10] Stack M.S., Johnson D.A. (1994). Human mast cell tryptase activates single-chain urinary-type plasminogen activator (pro-urokinase). J. Biol. Chem..

[11] Fajardo I., Pejler G. (2003). Human mast cell β-tryptase is a gelatinase. J. Immunol..

[12] Itoh Y., Sendo T., Oishi R. (2005). Physiology and pathophysiology of proteinase-activated receptors (PARs): Role of tryptase/PAR-2 in vascular endothelial barrier function. J. Pharmacol. Sci..

[13] Rickard A., Portell C., Kell P.J., Vinson S.M., McHowat J. (2005). Protease-activated receptor stimulation activates a Ca^2+^-independent phospholipase A2 in bladder microvascular endothelial cells. Am. J. Physiol. Ren. Physiol..

[14] Matej R., Mandàkovà P., Netikovà I., Poucková P., Olejár T. (2007). Proteinase-activated receptor-2 expression in breast cancer and the role of trypsin on growth and metabolism of breast cancer cell line MDA MB-231. Physiol. Res..

[15] Morris D.R., Ding Y., Ricks T.K., Gullapalli A., Wolfe B.L., Trejo J. (2006). Protease-activated receptor-2 is essential for factor VIIa and Xa-induced signaling, migration, and invasion of breast cancer cells. Cancer Res..

[16] Ammendola M., Leporini C., Marech I., Gadaleta C.D., Scognamillo G., Sacco R., Sammarco G., de sarro G., Russo E., Ranieri G. (2014). Targeting mast cells tryptase in tumor microenvironment: A potential antiangiogenetic strategy. BioMed Int. Res..

[17] Ammendola M., Sacco R., Sammarco G., Donato G., Zuccalà V., Luposella M., Patruno R., Marech I., Montemurro S., Zizzo N. (2014). Mast cells density positive to tryptase correlates with angiogenesis in pancreatic ductal adenocarcinoma patients having undergone surgery. Gastroenterol. Res. Pract..

[18] Donato G., Conforti F., Camastra C., Ammendola M., Donato A., Renzulli A. (2014). The role of mast cell tryptases in cardiac myxoma: Histogenesis and development of a challenging tumor. Oncol. Lett..

[19] Ammendola M., Zuccalà V., Patruno R., Russo E., Luposella M., Amorosi A., Vescio G., Sammarco G., Montemurro S., de Sarro G. (2013). Tryptase-positive mast cells and angiogenesis in keloids: A new possible post-surgical target for prevention. Updat. Surg..

[20] Ranieri G., Ammendola M., Patruno R., Celano G., Zito F.A., Montemurro S., Rella A., di Lecce V., Gadaleta C.D., de Sarro G. (2009). Tryptase-positive mast cells correlate with angiogenesis in early breast cancer patients. Int. J. Oncol..

[21] Ammendola M., Marech I., Sammarco G., Zuccalà V., Luposella M., Zizzo N., Patruno R., Crovace A., Ruggieri E., Zito A.F. (2015). Infiltrating mast cells correlate with angiogenesis in bone metastases from gastric cancer patients. Int. J. Mol. Sci..

[22] Ribatti D., Ranieri G. (2015). Tryptase, a novel angiogenic factor stored in mast cell granules. Exp. Cell Res..

[23] Malfettone A., Silvestris N., Saponaro C., Ranieri G., Russo A., Caruso S., Popescu O., Simone G., Paradiso A., Mangia A. (2013). High density of tryptase-positive mast cells in human colorectal cancer: A poor prognostic factor related to protease-activated receptor 2 expression. J. Cell. Mol. Med..

[24] Soreide K., Janssen E.A., Körner H., Baak J.P. (2006). Trypsin in colorectal cancer: Molecular biological mechanisms of proliferation, invasion, and metastasis. J. Pathol..

[25] Darmoul D., Marie J.C., Devaud H., Gratio V., Laburthe M. (2001). Initiation of human colon cancer cell proliferation by trypsin acting at protease-activated receptor-2. Br. J. Cancer.

[26] Uusitalo-Jarvinen H., Kurokawa T., Mueller B.M., Andrade-Gordon P., Friedlander M., Ruf W. (2007). Role of protease activated receptor 1 and 2 signaling in hypoxia-induced angiogenesis. Arterioscler. Thromb. Vasc. Biol..

[27] Liu Y., Mueller B.M. (2006). Protease-activated receptor-2 regulates vascular endothelial growth factor expression in MDA-MB-231 cells via MAPK pathways. Biochem. Biophys. Res. Commun..

[28] Yano H., Kinuta M., Tateishi H., Nakano Y., Matsui S., Monden T., Okamura J., Sakai M., Okamoto S. (1999). Mast cell infiltration around gastric cancer cells correlates with tumor angiogenesis and metastasis. Gastric Cancer.

[29] Sedda S., Marafini I., Caruso R., Pallone F., Monteleone G. (2014). Proteinase activated-receptors-associated signaling in the control of gastric cancer. World J. Gastroenterol..

[30] Ribatti D., Guidolin D., Marzullo A., Nico B., Annese T., Benagiano V., Crivellato E. (2010). Mast cells and angiogenesis in gastric carcinoma. Int. J. Exp. Pathol..

[31] Wang G.J., Wang Y.B., Li D.N., Deng B.B. (2013). Expression of protease-activated receptor-2 in human gastric stromal tumor and its clinic-pathological significance. Hepatogastroenterology.

[32] Zhang C., Gao G.R., Lv C.G., Zhang B.L., Zhang Z.L., Zhang X.F. (2012). Protease-activated receptor-2 induces expression of vascular endothelial growth factor and cyclooxygenase-2 via the mitogen-activated protein kinase pathway in gastric cancer cells. Oncol. Rep..

[33] Washington K. (2010). 7th Edition of the AJCC Cancer Staging Manual: Stomach. Ann. Surg. Oncol..

[34] Geng Y., Chen X., Qiu J., Zhou Y., Wang J., Liu L., Shao Y., Yin Y. (2014). Human epidermal growth factor receptor-2 expression in primary and metastatic gastric cancer. Int. J. Clin. Oncol..

[35] Wang X., Chen X., Fang J., Yang C. (2013). Overexpression of both VEGF-A and VEGF-C in gastric cancer correlates with prognosis, and silencing of both is effective to inhibit cancer growth. Int. J. Clin. Exp. Pathol..

[36] Zhao Y., Wu K., Cai K., Zhai R., Tao K., Wang G., Wang J. (2012). Increased numbers of gastric-infiltrating mast cells and regulatory T cells are associated with tumor stage in gastric adenocarcinoma patients. Oncol. Lett..

[37] Mukherjee S., Bandyopadhyay G., Dutta C., Bhattacharya A., Karmakar R., Barui G. (2009). Evaluation of endoscopic biopsy in gastric lesions with a special reference to the significance of mast cell density. Indian J. Pathol. Microbiol..

[38] Bhattacharyya S.P., Drucker I., Reshef T., Kirshenbaum A.S., Metcalfe D.D., Mekori Y.A. (1998). Activated T lymphocytes induce degranulation and cytokine production by human mast cells following cell-to-cell contact. J. Leukoc. Biol..

[39] Marech I., Ammendola M., Sacco R., Sammarco G., Zuccalà V., Zizzo N., Leporini C., Luposella M., Patruno R., Filippelli G. (2016). Tumor-associated macrophages correlate with microvascular bed extension in colorectal cancer patients. J. Cell. Mol. Med..

[40] Ammendola M., Patruno R., Sacco R., Marech I., Sammarco G., Zuccalà V., Luposella M., Zizzo N., Gadaleta C., Porcelli M. (2016). Mast cells positive to tryptase and tumor-associated macrophages correlate with angiogenesis in locally advanced colorectal cancer patients undergone to surgery. Expert Opin. Ther. Targets.

[41] Patruno R., Marech I., Zizzo N., Ammendola M., Nardulli P., Gadaleta C., Introna M., Capriuolo G., Rubini R.A., Ribatti D. (2014). C-Kit expression, angiogenesis, and grading in canine mast cell tumor: A unique model to study c-Kit driven human malignancies. BioMed Res. Int..

[42] De Souza Junior D.A., Santana A.C., da Silva E.Z., Oliver C., Jamur M.C. (2015). The Role of Mast Cell Specific Chymases and Tryptases in Tumor Angiogenesis. BioMed Res. Int..

[43] Wasiuk A., de Vries V.C., Hartmann K., Roers A., Noelle R.J. (2009). Mast cells as regulators of adaptive immunity to tumors. Clin. Exp. Immunol..

[44] Norrby K. (2002). Mast cells and angiogenesis. APMIS.

[45] Visciano C., Prevete N., Liotti F., Marone G. (2015). Tumor-Associated Mast Cells in Thyroid Cancer. Int. J. Endocrinol..

[46] Marone G., Varricchi G., Loffredo S., Granata F. (2015). Mast cells and basophils in inflammatory and tumor angiogenesis and lymphangiogenesis. Eur. J. Pharmacol..

[47] Loffredo S., Staiano R.I., Granata F., Genovese A., Marone G. (2014). Immune cells as a source and target of angiogenic and lymphangiogenic factors. Chem. Immunol. Allergy.

[48] Zhang X., Wang W., Mize G.J., Takayama T.K., True L.D., Vessella R.L. (2013). Protease-activated receptor 2 signaling up regulates angiogenic growth factors in renal cell carcinoma. Exp. Mol. Pathol..

[49] Rasmussen J.G., Riis S.E., Frobert O., Yang S., Kastrup J., Zachar V., Simonsen U., Fink T. (2012). Activation of protease-activated receptor 2 induces VEGF independently of HIF-1. PLoS ONE.

[50] Chang L.H., Pan S.L., Lai C.Y., Tsai A.C., Teng C.M. (2013). Activated PAR-2 regulates pancreatic cancer progression through ILK/HIF-α-induced TGF-α expression and MEK/VEGF-A-mediated angiogenesis. Am. J. Pathol..

[51] Ammendola M., Sacco R., Sammarco G., Piardi T., Zuccalà V., Patruno R., Zullo A., Zizzo N., Nardo B., Marech I. (2016). Mast Cells positive to tryptase, endothelial cells positive to protease-activated receptor-2, and microvascular density correlate among themselves in hepatocellular carcinoma patients who have undergone surgery. Onco. Targets Ther..

[52] Hirakawa S. (2009). From tumor lymphangiogenesis to lymphvascular niche. Cancer Sci..

[53] Wissman C., Detmar M. (2006). Pathways targeting tumor lymphangiogenesis. Clin. Cancer Res..

[54] Liu X., Cai H., Shi Y., Wang Y. (2012). Prognsotic factors in patients with node-negative gastric cancer: A single center experience from China. J. Gastrointest. Surg..

[55] Sjo O.H., Merok M.A., Svindland A., Nesbakken A. (2012). Prognostic impact of lymph node harvest and lymph node ratio in patients with colon cancer. Dis. Colon Rectum.

[56] Nam E.S., Kim D.H., Jang G.T., Park H.R., Kim J.R., Shin H.S. (2002). Correlation of Mast Cell Densities, Angiogenesis and Vascular Endothelial Growth Factor in Proper Muscle Gastric Carcinomas. Cancer Res. Treat..

[57] Micu G.V., Staniceanu F., Sticalru L.C., Popp C.G., Bastian A.E., Gramada E., Pop G., Mateescu R.B., Rimbaş M., Archip B. (2016). Correlations between the density of tryptase positive mast cells (DMTC) and that of new blood vessels (CD105^+^) in patients with gastric cancer. Rom. J. Intern. Med..

[58] Ammendola M., Sacco R., Sammarco G., Luposella M., Patruno R., Gadaleta C.D., de Sarro G., Ranieri G. (2016). Mast Cell-Targeted Strategies in Cancer Therapy. Transfus Med. Hemother..

[59] Erba F., Fiorucci L., Pascarella S., Menegatti E., Ascenzi P., Ascoli F. (2001). Selective inhibition of human mast cell tryptase by gabexate mesylate, an antiproteinase drug. Biochem. Pharmacol..

[60] Mori S., Itoh Y., Shinohata R., Sendo T., Oishi R., Nishibori M. (2003). Nafamostat mesilate is an extremely potent inhibitor of human tryptase. J. Pharmacol. Sci..

[61] Humbert M., Castéran N., Letard S., Hanssens K., Iovanna J., Finetti P., Bertucci F., Bader T., Mansfield C.D., Moussy A. (2010). Masitinib combined with standard gemcitabine chemotherapy: in vitro and in vivo studies in human pancreatic tumor cell lines and ectopic mouse model. PLoS ONE.

[62] Marech I., Patruno R., Zizzo N., Gadaleta C., Introna M., Zito A.F., Gadaleta C.D., Ranieri G. (2013). Masitinib (AB1010), from canine tumor model to human clinical development: Where we are?. Crit. Rev. Oncol. Hematol..

[63] Deplanque G., Demarchi M., Hebbar M., Flynn P., Melichar B., Atkins J., Nowara E., Moyé L., Piquemal D., Ritter D. (2015). A randomized, placebo-controlled phase III trial of masitinib plus gemcitabine in the treatment of advanced pancreatic cancer. Ann. Oncol..

[64] Tamura S., Takeno A., Miki H. (2011). Lymph node dissection in curative gastrectomy for advanced gastric cancer. Int. J. Surg. Oncol..

[65] Verlato G., Giacopuzzi S., Bencivenga M., Morgagni P., de Manzoni G. (2014). Problems faced by evidence-based medicine in evaluating lymphadenectomy for gastric cancer. World J. Gastroenterol.

[66] Ranieri G., Grammatica L., Patruno R., Zito A.F., Valerio P., Iacobellis S., Gadaleta C., Gasparini G., Ribatti D. (2007). A possible role of thymidine phosphorylase expression and 5-fluorouracil increased sensitivity in oropharyngeal cancer patients. J. Cell. Mol. Med..

